# Vascular endothelial growth factors and angiopoietins as new players in mastocytosis

**DOI:** 10.1007/s10238-021-00693-0

**Published:** 2021-03-09

**Authors:** Simone Marcella, Angelica Petraroli, Mariantonia Braile, Roberta Parente, Anne Lise Ferrara, Maria Rosaria Galdiero, Luca Modestino, Leonardo Cristinziano, Francesca Wanda Rossi, Gilda Varricchi, Massimo Triggiani, Amato de Paulis, Giuseppe Spadaro, Stefania Loffredo

**Affiliations:** 1 Department of Translational Medical Sciences, University of Naples Federico II, 80131 Naples, Italy; 2 Center for Basic and Clinical Immunology Research (CISI), University of Naples Federico II, 80131 Naples, Italy; 3 World Allergy Organization (WAO) Center of Excellence, 80131 Naples, Italy; 4 Division of Allergy and Clinical Immunology, University of Salerno, 84084 Fisciano, SA Italy; 5 Institute of Experimental Endocrinology and Oncology (IEOS), National Research Council, 80131 Naples, Italy

**Keywords:** Angiopoietins, Mast cells, Mastocytosis, Stem cell factor, Tryptase, Vascular endothelial growth factors

## Abstract

Mastocytosis is a disorder characterized by the abnormal proliferation and/or accumulation of mast cells in different organs. More than 90% of patients with systemic mastocytosis have a gain-of-function mutation in codon 816 of the KIT receptor on mast cells (MCs). The symptoms of mastocytosis patients are related to the MC-derived mediators that exert local and distant effects. MCs produce angiogenic and lymphangiogenic factors, including vascular endothelial growth factors (VEGFs) and angiopoietins (ANGPTs). Serum concentrations of VEGF-A, VEGF-C, VEGF-D, ANGPT1 and ANGPT2 were determined in 64 mastocytosis patients and 64 healthy controls. Intracellular concentrations and spontaneous release of these mediators were evaluated in the mast cell lines ROSA^KIT WT^ and ROSA ^KIT D816V^ and in human lung mast cells (HLMCs). VEGF-A, ANGPT1, ANGPT2 and VEGF-C concentrations were higher in mastocytosis patients compared to controls. The VEGF-A, ANGPT2 and VEGF-C concentrations were correlated with the symptom severity. ANGPT1 concentrations were increased in all patients compared to controls. ANGPT2 levels were correlated with severity of clinical variants and with tryptase levels. VEGF-A, ANGPT1 and VEGF-C did not differ between indolent and advanced mastocytosis. ROSA^KIT WT^, ROSA^KIT D816V^ and HLMCs contained and spontaneously released VEGFs and ANGPTs. Serum concentrations of VEGFs and ANGPTs are altered in mastocytosis patients.

**Supplementary Information:**

The online version contains supplementary material available at 10.1007/s10238-021-00693-0.

## Introduction

Mastocytosis is a rare clonal disorder characterized by uncontrolled proliferation, abnormal accumulation and survival of mast cells in several organs [1, 2]. This pathological condition is due to a somatic activating mutations of *KIT* gene that encodes for tyrosine kinase receptor KIT (CD117) largely expressed on mast cells [3, 4]. More than 90% of patients with systemic mastocytosis have a gain-of-function mutation in codon 816 of the receptor tyrosine kinase KIT, where a valine is substituted for an aspartate (*KIT* D816V) [5]. This mutation leads to an autophosphorylation of KIT receptor which results in MC proliferation also in the absence of the KIT ligand, stem cell factor (SCF) [5], which induces maturation, activation and proliferation of MCs [6, 7]. Mastocytosis is a heterogeneous group of neoplastic conditions [8] ranging from a skin-limited disorder [e.g., cutaneous mastocytosis (CM)] to severe forms involving multiple organs [e.g., systemic mastocytosis (SM)] [8, 9]. The symptoms of mastocytosis are the consequence of infiltration, activation and degranulation of MCs which exert local and systemic effects [10].

MCs are immune cells derived from bone marrow CD34^+^/CD117^+^ progenitors [11] which are localized in all tissues, including the skin [12], the mucosa of respiratory tract [13], the gastrointestinal system [14] and the heart [15]. Their number increases in several disorders such as bacterial, viral and parasitic infections [16, 17], allergic disorders [18, 19], arthritis [20], cardiovascular disorders [15, 21], cancer [22–24] and mastocytosis [10].

A plethora of stimuli (i.e., antigens, allergens, bacterial and viral superallergens, cytokines, chemokines) activates MCs to release two groups of inflammatory molecules: pre-formed mediators stored in their cytoplasmatic granules (e.g., histamine, α- and β-tryptase, chymase, and heparin) and de novo synthesized molecules (e.g., cytokines, chemokines, growth factors and lipid mediators) [25–27]. Immunological and non-immunological stimuli can induce the release of vascular endothelial growth factors (VEGFs) [28–34] and angiopoietins (ANGPTs) [35] from human MCs.

The VEGF family, in humans, consists of five separate gene products: VEGF-A, VEGF-B and placental growth factor (PlGF) are key regulators of physiological and pathological blood vessel growth [36, 37], whereas VEGF-C and VEGF–D modulate lymphangiogenesis [38]. VEGFs bind three tyrosine kinase receptors (VEGFR-1, -2, -3) expressed on blood (BEC) and lymphatic endothelial cells (LECs) [39]. VEGF-A, initially named vascular permeability factor (VPF), was discovered by Dvorak and collaborators for its permeabilizing activity [40]. It was found that VEGF-A is at least 50 times more potent than histamine in inducing vascular permeability [41, 42].

The ANGPT system is another pathway regulating vascular barrier functions [43]. In humans, ANGPT1 and ANGPT2 are the two primary angiopoietins: ANGPT1 is a vascular stabilizer acting on Tie2 receptor on BECs [44]. By contrast, ANGPT2 is an inhibitory ligand of the Tie2 receptor that disrupts the integrity of the blood vessel wall, thus counteracting vascular normalization [45].

Increased concentrations of circulating VEGFs and/or ANGPTs have been found in different human disorders characterized by increased vascular permeability or angiogenesis such as cardiovascular diseases [39, 46–48], cancer [49, 50], systemic capillary leak syndrome [51], angioedema [52, 53] and sepsis [54–56].

The role of VEGFs and ANGPTs in the pathophysiology of different forms of mastocytosis has not been thoroughly investigated. The aim of this paper was to evaluate the serum concentrations of VEGF-A, VEGF-C, VEGF-D, ANGPT1 and ANGPT2 in patients with different variants of mastocytosis and the expression of angiogenic and lymphangiogenic factors in human MC lines with or without D816V mutation.

## Materials and methods

### Patients selection

In a retrospective study, we evaluated 64 Caucasian patients with mastocytosis (31 males and 33 females; age range: 21–79 years; median age 46 years) followed at the University of Naples Federico II and at the University of Salerno whose clinical characteristics are summarized in Table 1 and Supplementary Table 1. None of patients was treated for mastocytosis with anti-inflammatory, immunosuppressant or immunomodulatory drugs at the time of blood sampling. A total of 64 healthy controls (HC) (31 males and 33 females; age range: 29–70 years; median age 43 years) were recruited as control. Inclusion criteria for this study were: the absence of any pathological conditions at the time of enrollment; expression of written informed consent. Exclusion criteria were: the presence of any condition that, in the opinion of the investigators, could interfere with the completion of the study such as pregnancy. Mediator-related symptoms were classified according to severity and frequency as follows: 13 patients had grade 0 (no symptoms), 13 had grade 1 (mild/ infrequent: prophylaxis and or as needed therapy), 26 had grade 2 (moderate: kept under control with anti-mediator type drugs daily), and 12 had grade 3 (severe and frequent: not sufficiently controlled with therapy). None of the patients had grade 4 characterized by severe adverse events which require immediate therapy and hospitalization. The diagnosis and classification of mastocytosis were made according to the recommendation of the World Health Organization (WHO) on the histological examination of a skin biopsy for cutaneous mastocytosis (CM) and of bone marrow biopsy for systemic mastocytosis (SM) [9, 57]. Patients were divided according to cutaneous and/or systemic involvement and the severity and frequency of symptoms. The first group (indolent) included maculopapular cutaneous mastocytosis (MPCM) (*n* = 3), mastocytosis in the skin (MIS) (*n* = 4), and indolent systemic mastocytosis (ISM) (*n* = 38). The second group (advanced) included patients with smoldering systemic mastocytosis (*n* = 9), aggressive systemic mastocytosis (*n* = 7), systemic mastocytosis associated with hematologic disease (SM-AHD) (*n* = 2) and mast cell leukemia (MCL) (*n* = 1) [2, 8, 9]. The most common mutation of KIT receptor in patients with indolent and aggressive SM is *KIT* D816V [2, 58]. The search of *KIT* D816V mutation was performed in 30 patients. In 21 of those patients, the presence of *KIT* mutation was found and in 9 patients was not found. Many patients with provisional diagnosis of isolated skin mastocytosis refused to undergo bone marrow biopsy. Circulating concentrations of angiogenic (VEGF-A), lymphangiogenic (VEGF-C and VEGF-D) factors, and angiopoietins (ANGPT1 and ANGPT2) were assessed in all patients and controls.

**Table 1:** Characteristics of patients with mastocytosis compared to healthy donors

Characteristics	Healthy (*n* = 64)	Patients (*n* = 64)
Age (years range)	43 (29–70)	46 (21–79)
Gender Male (%)	31 (48%)	31 (48%)
Tryptase (µg/L)^a^	5.6 ± 3.3	99.5 ± 243.4^b^
Symptom Grading (%)		
0		13 (20%)
1	NA	13 (20%)
2		26 (41%)
3		12 (19%)
Indolent	NA	
MPCM		3 (5%)
MIS		4 (6%)
I SM		38 (60%)
Clinical variants(%)		
Advanced	NA	
SSM		9 (14%)
ASM		7 (11%)
SM-AHD		2 (3%)
MCL		1 (1%)
Skin lesions (%)	NA	30 (47%)
Anaphylaxis (%)	NA	19 (30%)
Urticaria (%)	NA	35 (55%)
Flushing (%)	NA	46 (72%)
Pruritus (%)	NA	51 (80%)

*ASM* aggressive systemic mastocytosis; *ISM:* indolent systemic mastocytosis; *MCL* mast cell leukemia; *MIS* mastocytosis in skin; *MPCM* maculopapular cutaneous mastocytosis; *SM-AHD* systemic mastocytosis associated with hematologic disease; *SSM* smoldering systemic mastocytosis; *NA* not applicable; Data were analyzed by t-test

^a^Data are expressed as median values ± SD

^b^*p* < 0.005

### Serum collection

The Ethics Committee of Campania ASL Napoli 3 Sud (protocol number 68863) approved that serum, obtained during routine diagnostics, could be used for research investigating the pathophysiology of mastocytosis. Written informed consent was obtained from both mastocytosis patients and HC according to the principles expressed in the Declaration of Helsinki. The blood samples were collected by a clean venepuncture. After centrifugation (2000 × *g*, 22 °C, 20 min), the serum was divided into aliquots and stored at − 80 °C until tested. We collected the data about the clinical manifestations at onset of mastocytosis and 30% of patients have an history of anaphylaxis at the onset of disease. The collection of the samples for measurement of all metabolites was performed at least after 3 months from anaphylactic episodes.

### Tryptase assay

Serum tryptase concentration was measured by fluoro-enzyme immune assay (FEIA) using Uni-CAP100 (Phadia Diagnostics AB, Uppsala, Sweden). This technique allows to measure both α-tryptase and β-tryptase.

### Culture of human mast cells

The human mast cell lines ROSA^KIT WT^ and ROSA^KIT D816V^ were a generous gift from Michel Arock (Laboratoire de Biologie et de Pharmacologie Appliquee, Ecole Normale Supérieure de Cachan) [59]. ROSA^KIT WT^ and ROSA^KIT D816V^ mast cell lines were cultured at the density of 4 × 10^5^ cells/mL with and without recombinant SCF (80 ng/mL) (Peprotech, London, UK), respectively, in IMDM (Microgem, Naples, Italy) supplemented with 0.3% bovine serum albumin (Microgem, Naples, Italy), 1% L-glutamine (Sigma-Aldrich St. Louis, MO, USA), 2% nonessential aminoacids (Microgem, Naples, Italy), 1% vitamins solution (Gibco-Thermo-Fisher Waltham, MA, USA), 1% insulin-transferrin-selenium (Thermo-Fisher Waltham, MA, USA), and 1% sodium pyruvate (Gibco-Thermo-Fisher Waltham, MA, USA), 1% antibiotic–antimycotic solution (Lonza, Basel, CH). ROSA^KIT WT^ and ROSA^KIT D816V^ cells were incubated in 25 cm^2^ flask at 37 °C and 5% CO_2_ and counted after 4 days of culture. Primary human mast cells were isolated from lung parenchyma of patients undergoing thoracic surgery for lung cancer and were purified (> 98%) by immunomagnetic selection, as previously described [21]. The study protocol involving the use of human blood cells was approved by the Ethics Committee of the University of Naples Federico II (Protocol number 301/12), and written informed consent was obtained from donors according to the principles expressed in the Declaration of Helsinki. For VEGFs and ANGPTs analysis, all types of mast cells were incubated (37 °C, 5% CO_2_, 24 h) in complete medium. At the end of the experiments, the cells were centrifuged (1000 × *g*, 4 °C, 5 min) and the supernatants were stored at − 80 °C for subsequent determination of mediators. The cellular pellets were lysated in Tryton X-100 0.1% (Sigma-Aldrich, Saint Louis, MO, USA) and stored at − 80 °C for subsequent determination of intracellular mediator content.

### ELISA assay

VEGF-A, VEGF-C, VEGF-D, ANGPT1 and ANGPT2 concentrations in serum, in supernatant and in cellular lysates of ROSA^KIT WT^ and ROSA^KIT D816V^ were measured using commercially available ELISA KITs (R&D System, Minneapolis, MN, USA) according to the manufacturer's instructions. The serum concentrations of these mediators from mastocytosis patients and HC were expressed as pg/mL. Intracellular and released mediators from ROSA^KIT WT^ and ROSA^KIT D816V^ were expressed as pg/10^6^ cells.

### Statistical analysis

Data were analyzed with the GraphPad Prism 5 software package. Data were tested for normality using the D'Agostino–Pearson normality test. If normality was not rejected at 0.05 significance level, we used parametric tests. Otherwise, for not-normally distributed data we used nonparametric tests. Statistical analysis was performed by unpaired two-tailed t-test or two-tailed Mann–Whitney test as indicated in figure legends. Correlations between two variables were assessed by Spearman’s correlation analysis and reported as coefficient of correlation (*r*). A *p* value ≤ 0.05 was considered statistically significant. Serum levels of VEGF-A, VEGF-C, VEGF-D, ANGPT1 and ANGPT2 are shown as the median (horizontal black line), the 25th and 75th percentiles (boxes) and the 5th and 95th percentiles (whiskers) of 64 controls and 64 patients.

## Results

### VEGF and ANGPT serum concentrations in patients with mastocytosis

**Fig. 1:**
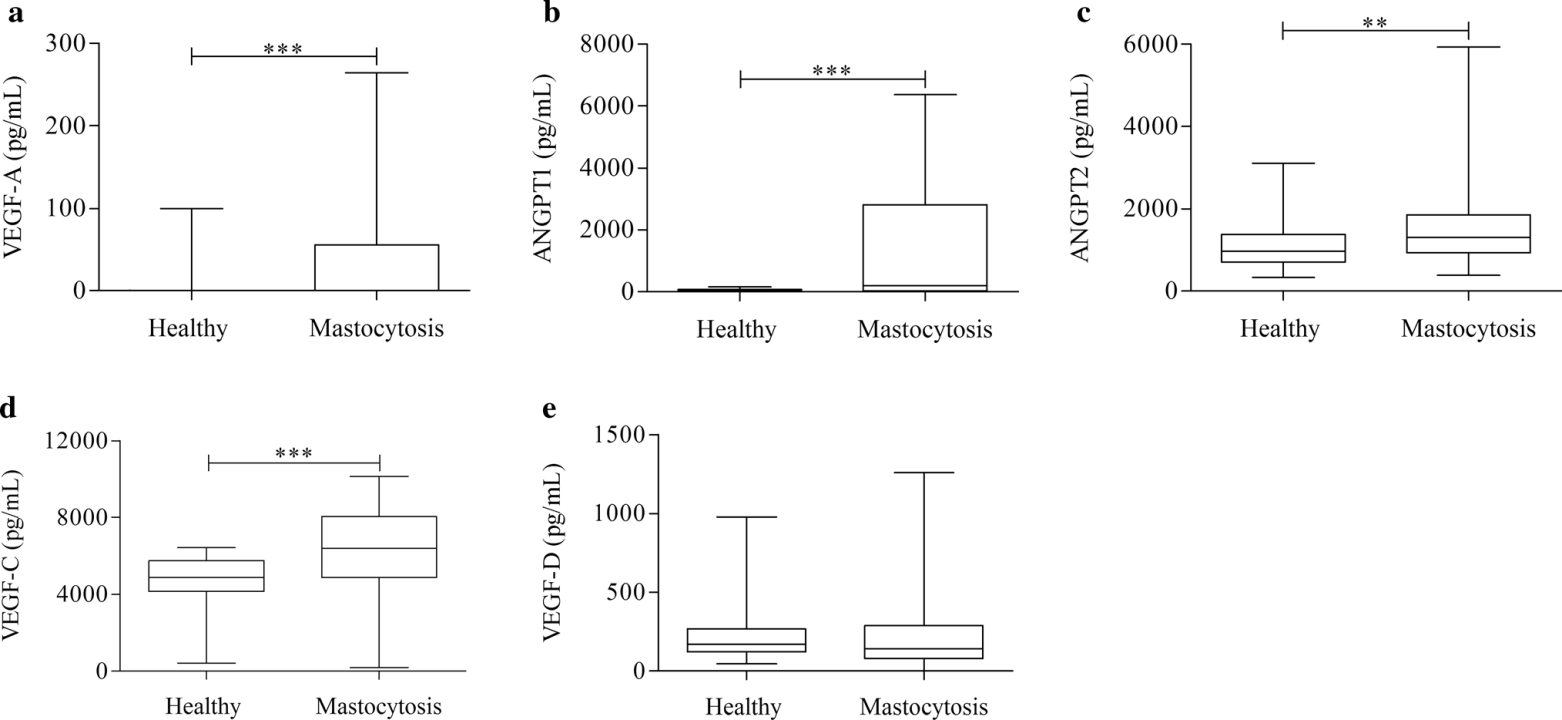
VEGF-A (**a**), ANGPT1 (**b**), ANGPT2 (**c**), VEGF-C (**d**) and VEGF-D (**e**) serum levels in healthy donors and in patients with mastocytosis. Data are shown as the median (horizontal black line), the 25th and 75th percentiles (boxes) and the 5th and 95th percentiles (whiskers) of 64 patients with mastocytosis and 64 healthy donors. **p < 0.01; ***p < 0.001

VEGF-A concentrations in mastocytosis patients have been studied only on small number of patients by Brockow et al. [60], whereas the ANGPTs levels have not yet been investigated. Therefore, in this study we evaluated the serum concentrations of VEGFs and ANGPTs in patients with mastocytosis (*N* = 64) compared to HC (*N* = 64). The characteristics of patients are reported in supplementary Table 1 (Supplementary Table 1). Figure 1a shows that VEGF-A serum levels of mastocytosis patients were higher than HC [VEGF-A: (34.63 ± 56.76) *vs* (9.21 ± 24.59) pg/mL]. Interestingly, both ANGPT2, which increases vascular permeability [61], and its antagonist ANGPT1 [62, 63] were increased in mastocytosis patients compared to HC [ANGPT1: (1,538 ± 2,136) *vs* (39.93 ± 45.60 pg/mL)] [ANGPT2: (1,492 ± 976) *vs* (1,085 ± 607) pg/mL] (Fig. 1b, c). In mastocytosis patients, the concentrations of different mediators did not correlate with each other (data not shown).

No data are available on the serum concentrations of lymphangiogenic factors in mastocytosis patients. Serum concentrations of VEGF-C were significantly higher in mastocytosis patients compared to the control group [VEGF-C: (6228 ± 2188) *vs* (4741 ± 1266) pg/mL] (Fig. 1d). Interestingly, the concentrations of VEGF-D, another lymphangiogenic factor, did not differ between the two groups [VEGF-D: (204.9 ± 213.1) *vs* (234.8 ± 184.5) pg/mL] (Fig. 1e). Surprisingly, VEGF-C and VEGF-D concentrations were positively correlated with each other (Supplementary Fig. 1).

There was no difference in VEGF and/or ANGPT concentrations between male and female values in both controls and patients (Supplementary Fig. 2). Moreover, the age of patients and the concentrations of the different mediators examined did not correlate (Supplementary Fig. 3, Additional File 4).

### Effects of disease severity on serum concentrations of VEGF and ANGPT in patients with mastocytosis

**Fig. 2:**
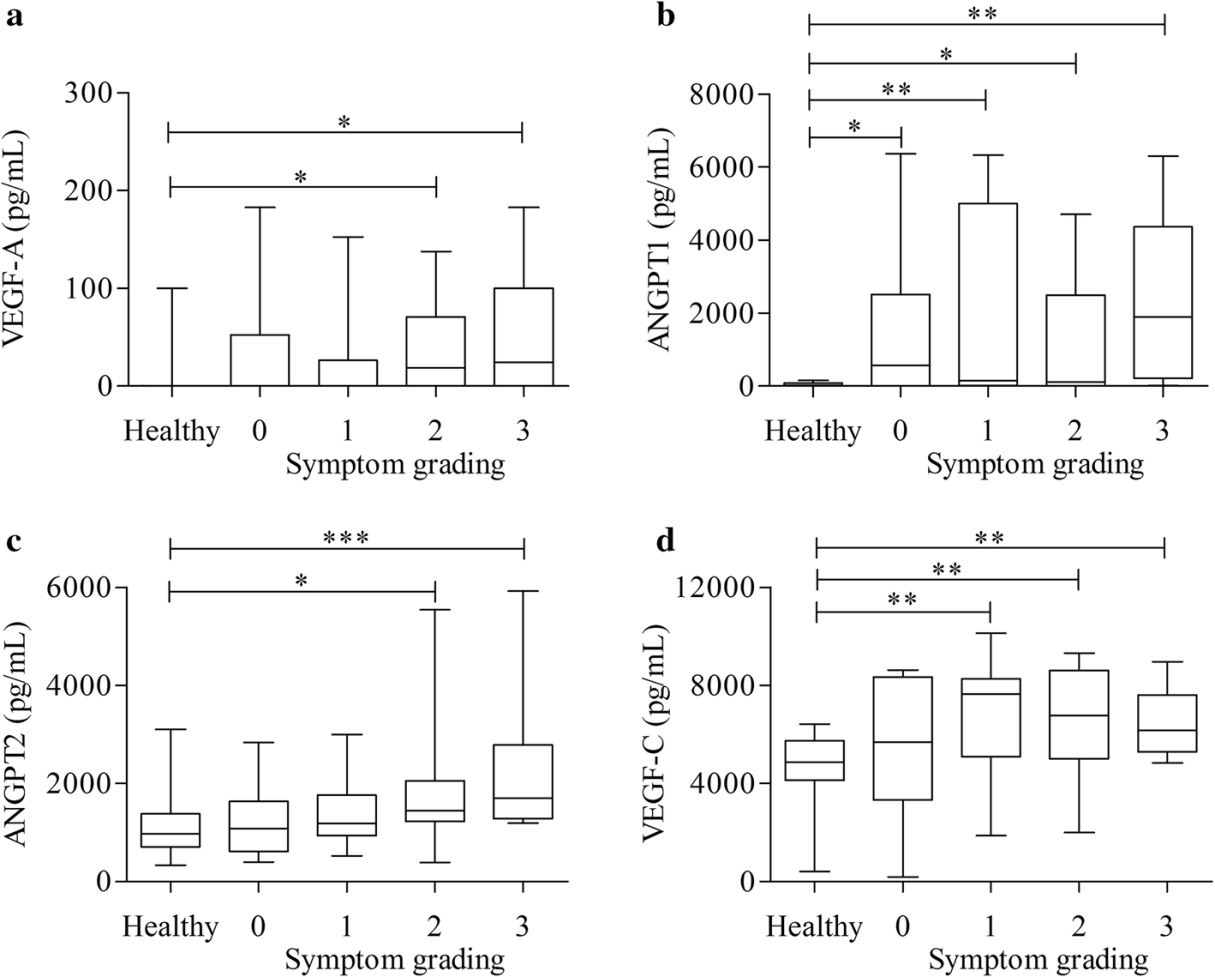
Effects of symptom grading on serum concentrations of VEGF-A (**a**), ANGPT1 (**b**), ANGPT2 (**c**) and VEGF-C (**d**). Serum levels of VEGFs and ANGPTs were measured in 13 patients with symptom grading 0, 13 patients with symptom grading 1, 20 patients with symptom grading 2 and 12 patients with symptom grading 3. *p < 0.05; **p < 0.01; ***p < 0.001

A triplex experimental analysis was used to verify whether enhanced levels of VEGF-A, ANGPT1, ANGPT2 and VEGF-C were correlated with mastocytosis severity. Mastocytosis patients were divided according to the severity of mediator-related symptoms, from grading 0 to grading 3 (see Materials and methods), and concentrations of VEGFs and ANGPTs were compared among groups. VEGF-A, ANGPT2 and VEGF-C levels were not increased in asymptomatic patients (grading 0) compared to controls (Fig. 2a, c, d). Symptomatic patients (grading 1 to 3) had elevated VEGF-C concentrations compared to HC (Fig. 2d); conversely, VEGF-A and ANGPT2 were altered only in symptomatic patients with grading 2 and 3 (Fig. 2a, c). Interestingly, ANGPT1 levels were increased in both asymptomatic and symptomatic patients compared to HC (Fig. 2b).

**Fig. 3:**
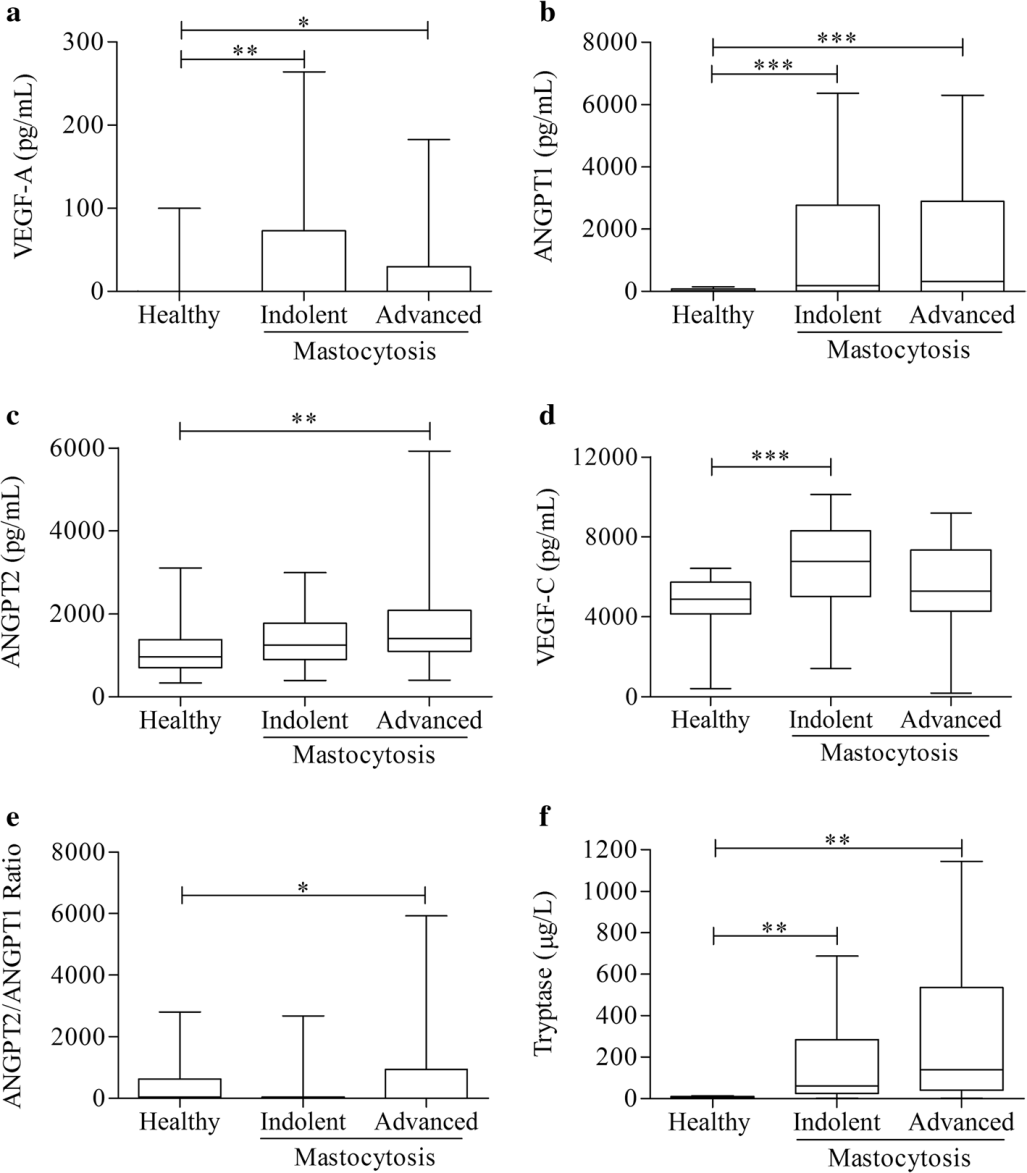
Effects of clinical variants of mastocytosis on serum concentrations of VEGF-A, VEGF-C, ANGPT1, ANGPT2, ANGPT2/ANGPT1 ratio, tryptase. VEGF-A (**a**), ANGPT1 (**b**), ANGPT2 (**c**), VEGF-C (**d**), ANGPT2/ANGPT1 (**e**) and tryptase (**f**) serum levels were determined in 64 healthy controls, in 45 patients with indolent variants and in 19 patients with advanced variants. *p < 0.05; **p < 0.01; ***p < 0.001

We also grouped patients according to their clinical variants in two groups (see Materials and methods): indolent (MPCM/MIS/ISM) and advanced (SSM/SM-AHD/ASM/MCL) mastocytosis. Figure 3 shows that VEGF-A (panel A) and ANGPT1 (panel B) concentrations did not differ between patients with indolent and advanced variants, but were altered in both groups when compared to controls. ANGPT2 levels, like tryptase (panel F), were higher in patients with advanced mastocytosis compared to indolent variants (panel C). Interestingly, VEGF-C was increased only in indolent mastocytosis compared to controls (panel D). The ANGPT2/ANGPT1 ratio (an index of vascular permeability) [53] was also increased in more severe patients (panel E).

**Fig. 4:**
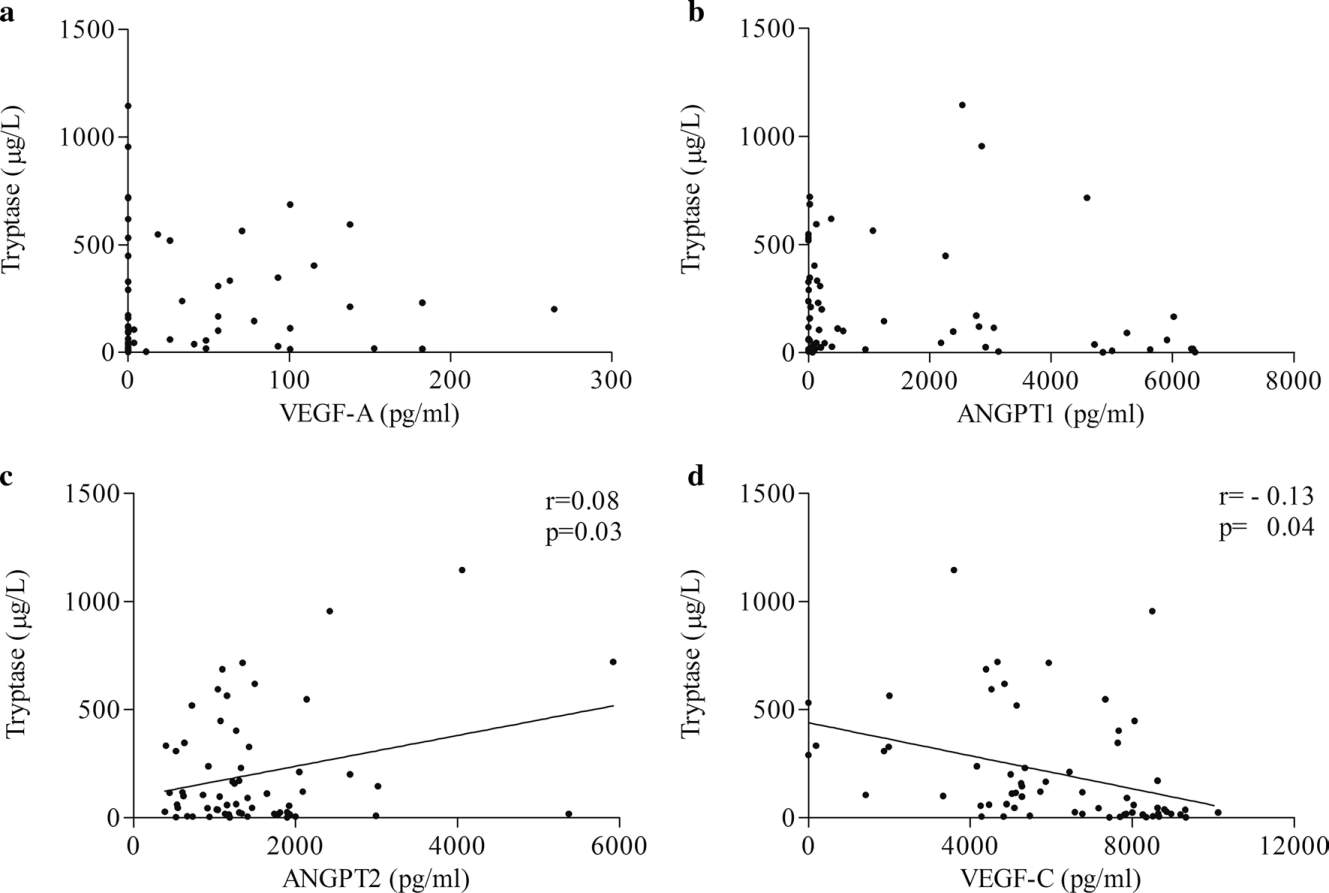
Correlations between VEGF-A, ANGPT1, ANGPT2, VEGF-C and tryptase concentrations. Correlations between two variables: VEGF-A and tryptase (**a**), ANGPT1 and tryptase (**b**), ANGPT2 and tryptase (**c**) and VEGF-C and tryptase (**d**) were assessed by Spearman’s correlation analysis and reported as coefficient of correlation (*r*). *p* < 0.05 was considered statistically significant

Patients with advanced forms of mastocytosis have elevated tryptase levels compared with those with indolent forms [64, 65]. Thus, we analyzed the correlations between VEGFs/ANGPTs and this important marker of mast cell activation/proliferation. The concentrations of VEGF-A (Fig. 4a) and ANGPT1 (Fig. 4b) were not correlated with tryptase levels. The concentrations of ANGPT2 positively correlated with tryptase concentrations (Fig. 4c). By contrast, the serum levels of VEGF-C negatively correlated with tryptase concentrations (Fig. 4d).

### Angiogenic and lymphangiogenic factors in primary human mast cells and in human mast cell lines

We have previously reported that VEGF-A, VEGF-C, and VEGF-D can be detected by immunohistochemistry in human lung mast cells [66]. The ROSA^KIT WT^ is a SCF-dependent human MC line expressing the high affinity receptor for IgE (FcεRI) [59]. The most frequent mutation affecting patients with mastocytosis is the D816V [5, 67]. The transfection with *KIT* D816V converted ROSA^KIT WT^ cells into an SCF-independent clone, ROSA^KIT D816V^, which produced a mastocytosis-like disease in mice [59].

**Fig. 5:**
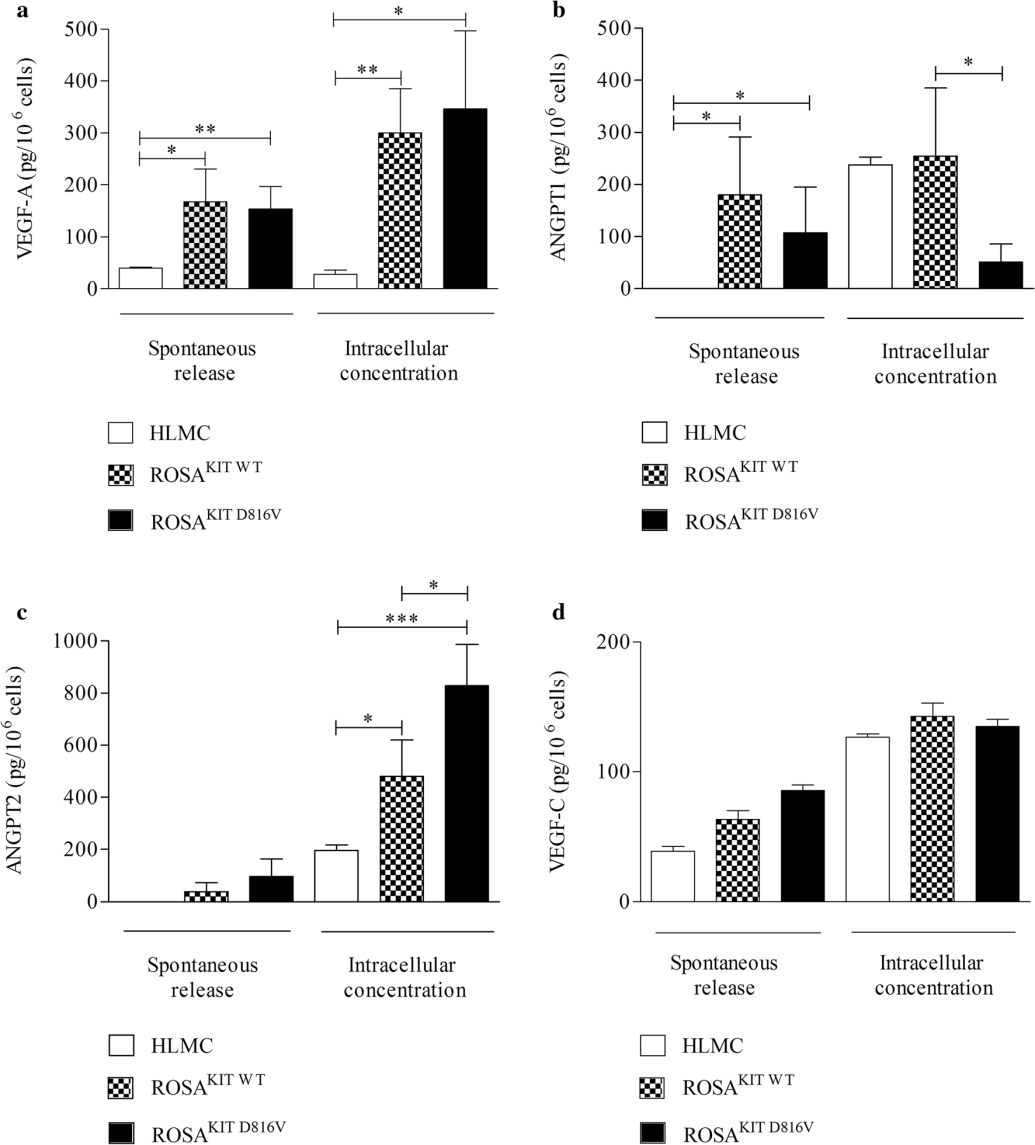
Spontaneous release and intracellular concentrations of VEGF-A (**a**), ANGPT1 (**b**), ANGPT2 (**c**), and VEGF-C (**d**) from human lung mast cells (HLMC), ROSA^KIT WT^ and ROSA^KIT D816^. ROSA^KIT WT^ and ROSA^KIT D816V^ were incubated (5% CO2, 37 °C, 24 h) with and without SCF (80 ng/ml), respectively. Data are the mean ± SD of 5 independent experiments. *p < 0.05; *p < 0.01; ***p < 0.001

We analyzed the basal content of VEGFs and ANGPTs in ROSA^KIT WT^ and ROSA^KIT D816V^ and also primary human MC derived from lung tissue (HLMCs). Figure 5 shows that both ROSA^KIT WT^ and ROSA^KIT D816V^ spontaneously released a large amount of VEGF-A (panel A), ANGPT1 (panel B), ANGPT2 (panel C) and VEGF-C (panel D). The spontaneous release of these mediators did not differ between the two mast cell lines. HLMCs release lower concentration of VEGF-A and ANGPT1 compared ROSA cells; by contrast, the release of ANGPT2 and VEGF-C did not differ among the three different human MCs.

ROSA^KIT WT^ and ROSA^KIT D816V^ contained VEGF-A (Fig. 5a), ANGPT1 (Fig. 5b), ANGPT2 (Fig. 5c) and VEGF-C (Fig. 5d). The content of VEGF-A and of VEGF-C was similar between wild type and mutated MC lines (Fig. 5a, d). ANGPT1 in lysates of ROSA^KIT WT^ was higher than in ROSA^KIT D816V^. By contrast, the intracellular content of ANGPT2 was higher in ROSA^KIT D816V^ than in ROSA^KIT WT^ (Fig. 5b, c). HLMCs contained lower levels of VEGF-A and ANGPT2 compared to ROSA cells.

## Discussion

Serum concentrations of VEGF-A, VEGF-C, ANGPT1 and ANGPT2 are increased in patients with mastocytosis compared to healthy controls. Some of these mediators such as VEGF-A [21, 31–34], VEGF-C [21, 29] and ANGPT1 and ANGPT2 [35] are expressed by human primary and neoplastic (e.g., LAD2, HMC-1) mast cells. There is a clinical correlation between the severity of mastocytosis and the plasma levels of these mast cell-derived mediators. In fact, circulating levels of VEGF-A, ANGPT2 and VEGF-C are increased in symptomatic, but not asymptomatic mastocytosis patients. Interestingly, the serum concentration of ANGPT1, which is mainly produced by pericytes and inhibits endothelial cell permeability [63], is increased in all mastocytosis patients.

The angiopoietin (ANGPT) family is an important group of factors, specific for vascular endothelium, whose functions are mediated through two tyrosine kinase receptors, Tie1 and Tie2 [43, 63]. The ANGPT-Tie ligand–receptor system exerts a key role in regulating vascular integrity [68, 69]. Besides their roles in the regulation of angiogenesis [62, 70] and lymphangiogenesis [71, 72], ANGPTs also modulate inflammation in several disorders [39, 48, 69, 73]. ANGPT1, produced by peri-endothelial mural cells (pericytes) [74] and immune cells [35, 75], is a potent agonist of Tie2 receptor on endothelial cells [44, 70]. ANGPT1 is an anti-inflammatory molecule that maintains vascular integrity [68, 76, 77]. ANGPT2, stored in Weibel–Palade bodies in endothelial cells [78], is considered a pro-inflammatory molecule [61, 79]. ANGPT2 inhibits ANGPT1/Tie2 interaction [62, 63], resulting in vascular instability and leakage [61].

It has been demonstrated that ANGPT1 inhibits the in vitro activation of the mouse mastocytoma cell line P815 and experimental anaphylactic shock in mice [80]. Anaphylaxis and anaphylactoid reactions are more frequent in mastocytosis patients compared to the general population [81]. It is possible to speculate that the increase in circulating ANGPT1 in all patients with mastocytosis might represent a protective factor in counterbalancing the vasopermeability effect of VEGF-A [41, 42, 82] and ANGPT2 [61].

Tryptase is a serine protease highly expressed by human mast cells and to a minor extent by basophils [83, 84]. Measurements of tryptase levels in serum have been used to assess mast cell load in systemic mastocytosis [64, 85–87]. In this study, we found that serum concentrations of tryptase are increased in indolent and advanced mastocytosis. Tryptase concentrations are positively correlated with the circulating levels of ANGPT2 and negatively correlated with VEGF-C. The latter observation is difficult to reconcile because there is evidence that activated human mast cells release both tryptase and VEGF-C [21, 39]. Moreover, tryptase serum concentrations are not correlated with the levels of VEGF-A and ANGPT1. Again, these results are rather unexpected because VEGF-A [28–34] and ANGPT1 [35] are expressed by human mast cells. However, many other immune and non-immune cells can produce and release VEGF-A [88–90] and ANGPT1 [63].

This study also examined the differential expression of several mediators in indolent and advanced mastocytosis. There is compelling evidence that mastocytosis is a heterogeneous condition with strikingly different prognostic profiles [2, 91]. Serum concentrations of tryptase, VEGF-A and ANGPT1 are increased in indolent and advanced mastocytosis compared to healthy controls. However, the lack of correlation between tryptase and both VEGF-A and ANGPT1 might indicate that alternative sources of the two latter mediators are involved in mastocytosis. This observation suggests that there are complex cellular and biochemical alterations in mastocytosis, in addition to the proliferation of mast cells.

ANGPT2, which is released mainly by endothelial cells [78], and ANGPT1/ANGPT2 ratio, an index of vascular permeability [53], are increased only in advanced mastocytosis. We found that serum concentrations of ANGPT2 are correlated with those of tryptase. The latter correlation might indicate that in patients with advanced mastocytosis these mediators are mainly derived from activated mast cells. These results are in line with those of our previous work in which we demonstrated that an endothelial dysfunction is detectable in patients with mastocytosis and is more severe in patients with high tryptase levels and advanced disease. Endothelial function appears to be negatively influenced by MC proliferation rather than by the severity of mediator-related symptoms [92].

VEGF-C and VEGF-D are the most important modulators of inflammatory and tumor lymphangiogenesis [93, 94] acting on VEGF receptor 3 (VEGFR-3) on LECs [38, 95]. These factors can be detected [66] and can be produced by activated human mast cells [21, 39]. Our results indicate that the serum concentrations of VEGF-C but not VEGF-D are markedly increased in patients with mastocytosis compared to healthy controls. The differential alterations of VEGF-C and VEGF-D in these patients are intriguing but not surprising. Recent evidence indicates that VEGF-C and VEGF-D can differently modulate the immune system [93]. The possible role of VEGF-C in mastocytosis deserves further investigations.

Human mast cells constitutively express VEGF receptors and Tie receptors for ANGPTs [28, 29, 35]. These receptors are functionally active because VEGFs [29] and ANGPT1 exert a chemotactic effect on human mast cells [35]. In this scenario, one may envisage a novel autocrine-loop involving angiogenic factors (i.e., VEGFs, ANGPTs) and their receptors on mast cells. In fact, VEGFs and ANGPT1 released by activated mast cells might attract progenitors of these cells to sites of neoplastic growth through the engagement of VEGFs and Tie2 receptors, respectively.

There is compelling evidence that human mast cells are a major source of several canonical (VEGF-A, ANGPTs) [21, 31–35] and non-canonical angiogenic factors (LTC_4_, LTD_4_, tryptase) [96, 97]. Valent and collaborators demonstrated that bone marrow microvessel density (MVD) is increased in patients with mastocytosis [98]. Moreover, BM MVD was significantly higher in systemic mastocytosis compared to cutaneous mastocytosis and healthy controls. Immunohistochemical staining revealed expression of VEGF-A in mast cell infiltrates. The same group of investigators extended the previous observation to canine mastocytosis by demonstrating the presence of VEGF in primary dog mastocytomas by immunohistochemistry and VEGF mRNA by PCR [99].

We have previously shown by immunohistochemistry that HLMCs contain VEGF-A, VEGF-C, and VEGF-D [66]. In this study, we confirm that immunoreactive VEGF-A is present in HLMCs and can be spontaneously released. We also examined the content and spontaneous release of several angiogenic factors in ROSA mast cell lines with (ROSA^KIT D816V^) and without *KIT* mutation (ROSA^KIT WT^) [59]. Both ROSA^KIT WT^ and ROSA^KIT D816V^ contained and spontaneously released VEGF-A, VEGF-C and ANGPT1. These findings agree with previous observations that the histamine content and FcεRI expression did not differ between both ROSA^KIT WT^ and ROSA^KIT D816V^ cell lines [59].

MicroRNA (miRNAs) are a large class of single-stranded RNA molecules that regulate a wide spectrum of cellular functions [100, 101]. Several miRNAs modulate different genes during mast cell activation [100, 102, 103] and the expression of pro- and anti-angiogenic factors [101, 104]. In particular, miR-221 and miR-222, highly expressed in endothelial cells, are upregulated during mast cell activation [102] and modulate the secretion of cytokines [105] and angiogenesis [104]. Therefore, it would be of interest to evaluate the expression of miR-221/222 in patients with different variants of mastocytosis. We cannot exclude the possibility that complex interplay between miR-221/222 and angiogenic/lymphangiogenic factors could contribute to the neoplastic progression of mastocytosis.

This study has a limitation that should be pointed out. Although more than 90% of patients with systemic mastocytosis have a mutation in codon 816 of KIT (*KIT* D816V) [5, 58], alternative *KIT* mutation in codon 816 (e.g., D816A/F/H/I/N/T/Y) has been described. In addition, to the tyrosine kinase domain (exons 17 and 18; e.g., D820G or N8221/K), at least 30 different *KIT* mutations have been identified in the extracellular (exon 8–9), transmembrane (exon 19; e.g., F522C) and juxtamembrane domains (exon 11; e.g., V560 G/I) in a small percentage of mastocytosis patients [106–109]. In this study, the identification of *KIT* D816V was not performed in all patients examined. In addition, other less common mutations were not investigated.

Mastocytosis is a heterogeneous group of neoplastic disorders characterized by complex pathology, distinct subtypes, and highly variable clinical courses [2, 8, 9]. Our findings indicate that VEGF and ANGPT concentrations are increased in patients with mastocytosis compared to controls. Several studies have shown that in addition to activating KIT mutations, additional mutations in other genes may occur in mastocytosis [106–108]. The contribution of *KIT* and other mutations to the altered production of VEGFs and ANGPTs in patients with different forms of mastocytosis remains to be investigated. In addition, further studies on larger cohorts of patients with different variants of mastocytosis could highlight the theragnostic significance of VEGF and ANGPT assays in these patients. Finally, classical and novel inhibitors of angiogenesis and/or lymphangiogenesis alone or in combination with other anti-neoplastic drugs are used in the treatment of cancer [63]. The current treatment options for patients with advanced mastocytosis need to be improved [2, 91]. Perhaps, the use of angiogenic/lymphangiogenic inhibitors could be considered for the treatment of selected patients with severe mastocytosis and high levels of circulating angiogenic factors.

## Supplementary Information

Below is the link to the electronic supplementary material.

Supplementary file1 (DOC 633 KB)

## Data Availability

All data generated or analyzed during this study are included in this published article and its supplementary information files.

## Abbreviations

ASM: Aggressive systemic mastocytosis
ANGPT: Angiopoietin
BEC: Blood endothelial cell
BM: Bone marrow
BSA: Bovine serum albumin
CM: Cutaneous mastocytosis
DCM: Diffuse cutaneous mastocytosis
FEIA: Fluoro-enzyme immune assay
HC: Healthy control
HLMC: Human lung mast cell
ISM: Indolent systemic mastocytosis
LEC: Lymphatic endothelial cell
MC: Mast cell
MCL: Mast cell leukemia
MCS: Mast cell sarcoma
MIS: Mastocytosis in skin
MPCM: Maculopapular cutaneous mastocytosis
MVD: Microvessel density
PlGF: Placental growth factor
SM-AHD: Systemic mastocytosis associated to hematologic disease
SSM: Smoldering systemic mastocytosis
SCF: Stem cell factor
SM: Systemic mastocytosis
Tie: Tyrosine kinase with immunoglobulin-like and EGF-like domains
VEGF: Vascular endothelial growth factor
VEGFR: Vascular endothelial growth factor receptor
VPF: Vascular permeability factor
