# Supplementary material for: Vascular endothelial growth factors and angiopoietins as new players in mastocytosis
Source: Clin Exp Med. 2021 Mar 9;21(3):415–27. doi: 10.1007/s10238-021-00693-0 (PMC8266723; doi:10.1007/s10238-021-00693-0)
Supplement: Supplementary file 1 — Supplementary file1 (DOC 633 KB) [file 10238_2021_693_MOESM1_ESM.doc]

**SUPPLEMENTARY INFORMATION**

**Supplementary Table 1** Characteristics of 64 adult patients with mastocytosis

| **Patient**  **No.** | **Sex** | **Age** | **Disease**  **Category** | **Tryptase**  **µg/L** | **Symptom**  **Grading** |
| --- | --- | --- | --- | --- | --- |
| **1** | F | 26 | ISM | 14.4 | 0 |
| **2** | F | 28 | MPCM | 5.7 | 0 |
| **3** | M | 49 | MPCM | 2.9 | 0 |
| **4** | M | 32 | ISM | 95.5 | 0 |
| **5** | M | 52 | ISM | 17.2 | 0 |
| **6** | F | 59 | ISM | 44.8 | 0 |
| **7** | M | 44 | ISM | 5.6 | 0 |
| **8** | F | 32 | ISM | 10.1 | 0 |
| **9** | M | 40 | MIS | 1.5 | 0 |
| **10** | M | 55 | ISM | 106 | 0 |
| **11** | F | 61 | ISM | 40.3 | 0 |
| **12** | F | 31 | ISM | 11.8 | 0 |
| **13** | M | 52 | ISM | 60 | 0 |
| **14** | M | 39 | ISM | 37.3 | 1 |
| **15** | M | 21 | MPCM | 5.8 | 1 |
| **16** | M | 47 | ISM | 66 | 1 |
| **17** | F | 44 | SSM | 46.1 | 1 |
| **18** | F | 57 | ISM | 59.2 | 1 |
| **19** | F | 43 | ISM | 17.7 | 1 |
| **20** | F | 35 | ISM | 46.1 | 1 |
| **21** | M | 44 | ISM | 9.6 | 1 |
| **22** | F | 50 | ISM | 24.4 | 1 |
| **23** | F | 36 | SM-AHD | 9.8 | 1 |
| **24** | M | 51 | ISM | 34.7 | 1 |
| **25** | F | 30 | MIS | 6.3 | 1 |
| **26** | F | 28 | ISM | 33.3 | 1 |
| **27** | M | 79 | ASM | 150 | 2 |
| **28** | F | 23 | ISM | 6.87 | 2 |
| **29** | M | 48 | SSM | 16.2 | 2 |
| **30** | M | 52 | ISM | 1.7 | 2 |
| **31** | M | 41 | ISM | 18.8 | 2 |
| **32** | M | 28 | ISM | 24.6 | 2 |
| **33** | M | 37 | SSM | 717 | 2 |
| **34** | M | 59 | ISM | 57.3 | 2 |
| **35** | F | 44 | SSM | 128 | 2 |
| **36** | M | 44 | ISM | 167 | 2 |
| **37** | F | 35 | ISM | 27.7 | 2 |
| **38** | F | 52 | ISM | 37.8 | 2 |
| **39** | M | 54 | ISM | 44.1 | 2 |
| **40** | M | 37 | MIS | 18 | 2 |
| **41** | F | 32 | MIS | 11.6 | 2 |
| **42** | M | 56 | ISM | 51.9 | 2 |
| **43** | F | 79 | SM-AHD | 54.8 | 2 |
| **44** | M | 43 | ASM | 23.8 | 2 |
| **45** | F | 48 | ISM | 21.2 | 2 |

Supplementary Table 1 (continued)

| **Patient**  **No.** | **Sex** | **Age** | **Disease**  **Category** | **Tryptase**  **µg/L** | **Symptom**  **Grading** |
| --- | --- | --- | --- | --- | --- |
| **46** | M | 59 | ISM | 68.7 | 2 |
| **47** | F | 48 | ISM | 56.4 | 2 |
| **48** | F | 34 | ISM | 30.8 | 2 |
| **49** | F | 54 | ISM | 59.4 | 2 |
| **50** | F | 41 | ISM | 32.8 | 2 |
| **51** | M | 69 | ASM | 532 | 2 |
| **52** | M | 40 | ASM | 290 | 2 |
| **53** | F | 59 | ASM | 720 | 3 |
| **54** | M | 76 | MCL | 1145 | 3 |
| **55** | M | 51 | ASM | 159 | 3 |
| **56** | M | 63 | SSM | 26.2 | 3 |
| **57** | F | 24 | SSM | 17.5 | 3 |
| **58** | F | 46 | ASM | 1.5 | 3 |
| **59** | F | 72 | ISM | 92 | 3 |
| **60** | F | 57 | SSM | 121 | 3 |
| **61** | F | 38 | ISM | 61.9 | 3 |
| **62** | M | 53 | SSM | 231 | 3 |
| **63** | F | 58 | ISM | 146 | 3 |
| **64** | F | 60 | SSM | 112 | 3 |

ASM: aggressive systemic mastocytosis; ISM: indolent systemic mastocytosis; MCL: mast cell leukemia; MIS: mastocytosis in skin; MPCM: macupapular coutaneous mastocytosis; SM-AHD: systemic mastocytosis associated with hematologic disease; SSM: smouldering systemic mastocytosis.


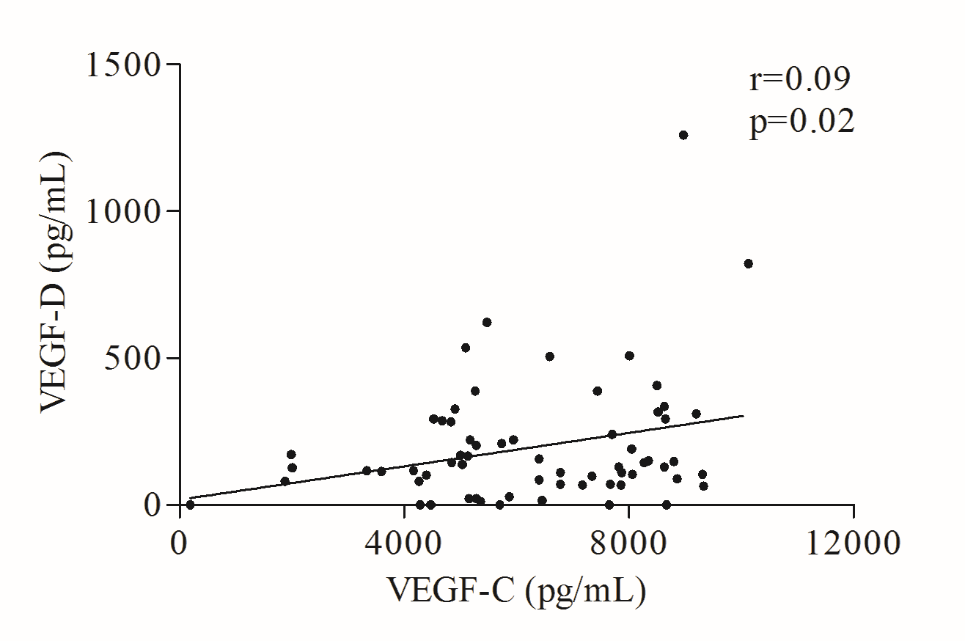


**Supplementary Fig. 1** Correlation between VEGF-C and VEGF-D serum levels in patients with mastocytosis. Correlation was assessed by Spearman’s correlation analysis and reported as coefficient of correlation (r). *p* < 0.05 was considered statistically significant.


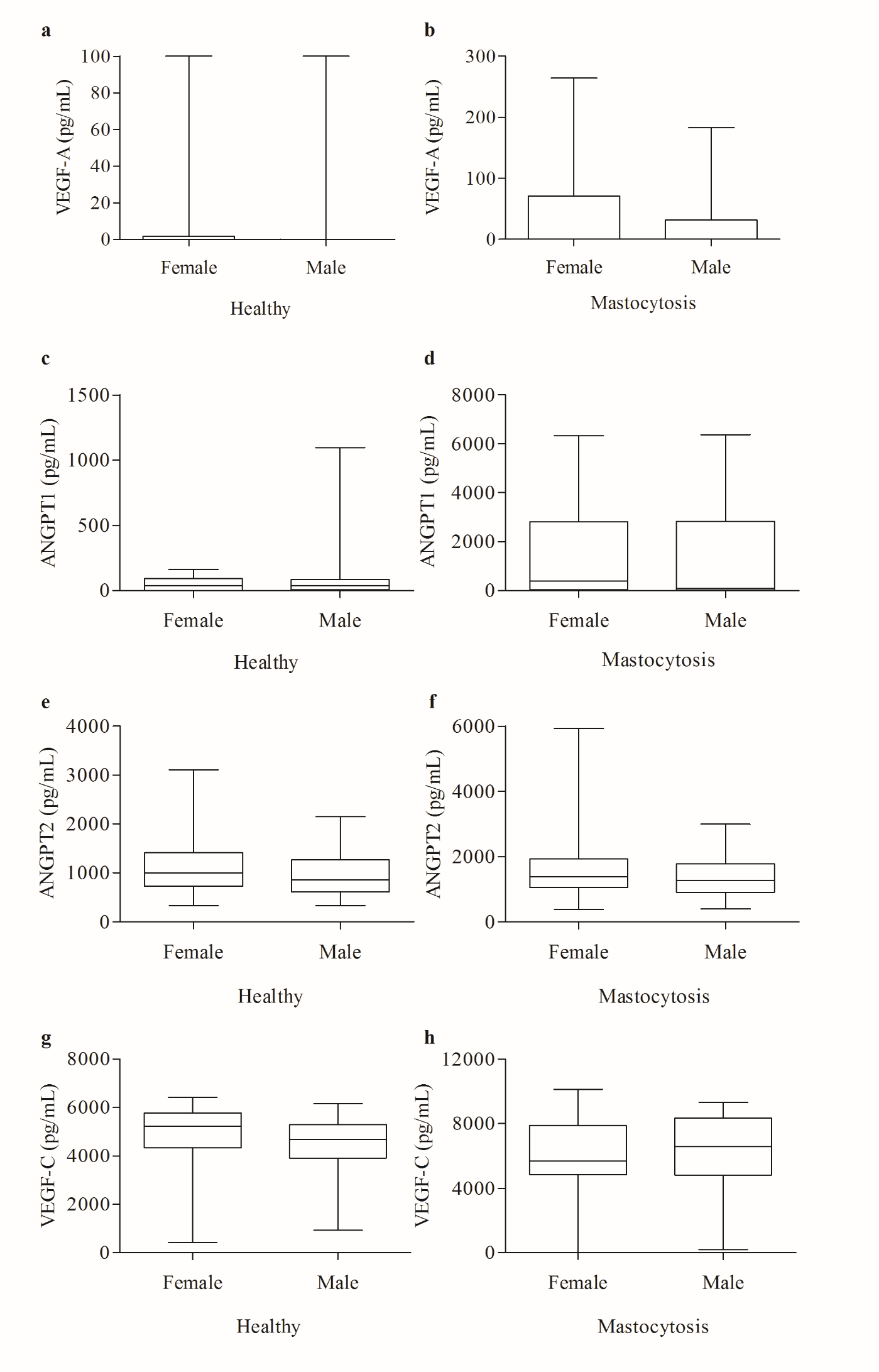


**Supplementary Fig. 2** Relationships between VEGF-A, ANGPT1, ANGPT2 and VEGF-C serum levels and gender of mastocytosis patients and healthy donors. VEGF-A (**a**-**b**), ANGPT1 (**c**-**d**), ANGPT2 (**e**-**f**) and VEGF-C (**g**-**h**) serum concentrations were measured in females (n=25) and males (n=22) healthy donors (**a**, **c**, **e**, **g**) and in females (n=33) and males (n=31) mastocytosis patients (**b**, **d**, **f**, **h**).


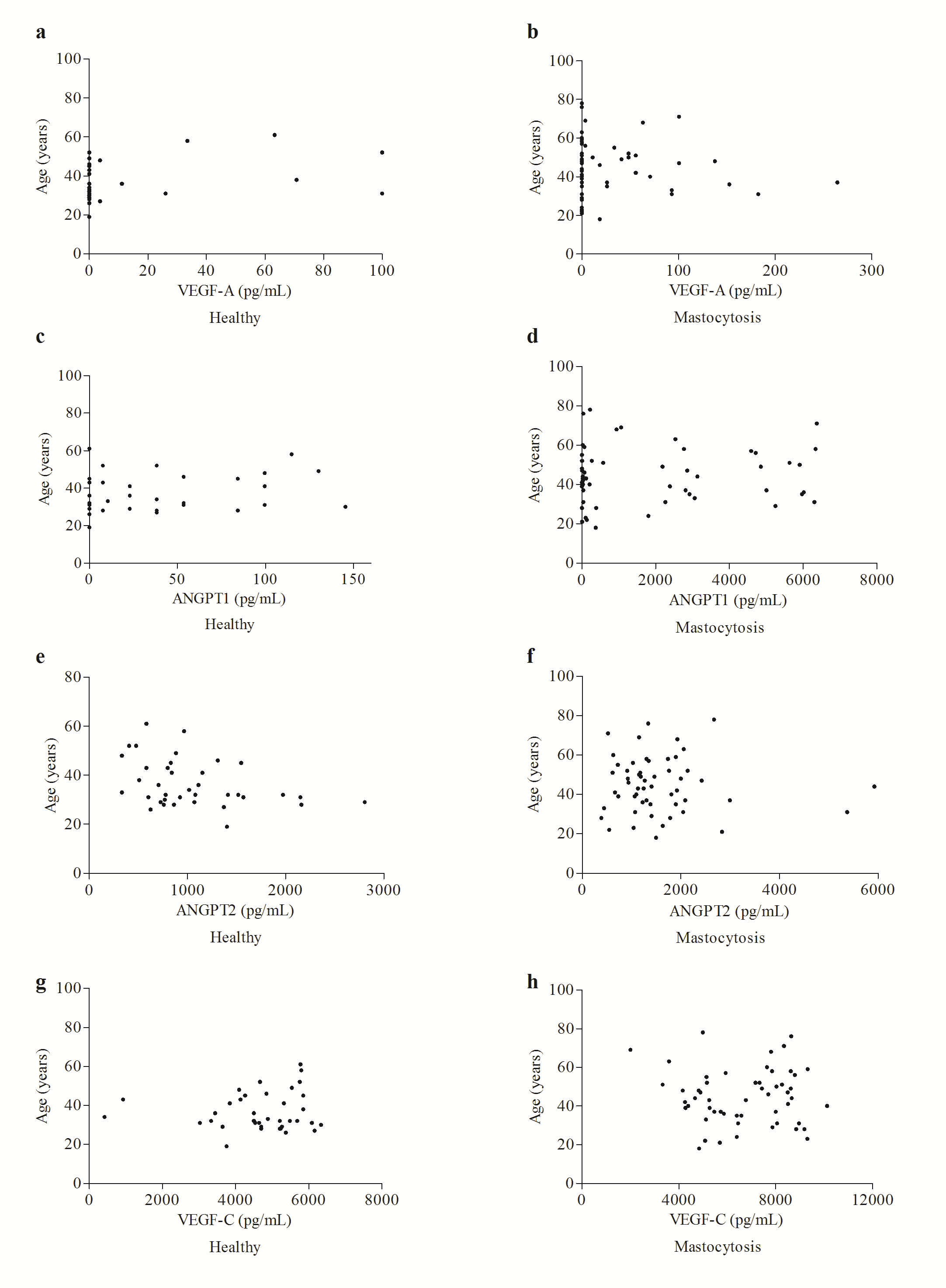


**Supplementary Fig. 3** Correlations between VEGF-A, ANGPT1, ANGPT2 and VEGF-C serum levels and age of patients affected by mastocytosis and healthy donors. VEGF-A (**a**-**b**), ANGPT1 (**c**-**d**), ANGPT2 (**e**-**f**) and VEGF-C (**g**-**h**) serum concentrations were measured in healthy donors (age range: 29-70 years; median age 43 years) (**a**, **c**, **e**, **g**) and in patients with mastocytosis (age range: 21–79 years; median age 46 years).Correlations between two variables: VEGF-A (**a**-**b**), ANGPT1 (**c**-**d**), ANGPT2 (**e**-**f**), VEGF-C (**g**-**h**) and age were assessed by Spearman’s correlation analysis and reported as coefficient of correlation (r)
